# Precision Motion Control of a Piezoelectric Actuator via a Modified Preisach Hysteresis Model and Two-Degree-of-Freedom H-Infinity Robust Control

**DOI:** 10.3390/mi14061208

**Published:** 2023-06-07

**Authors:** Ayad G. Baziyad, Irfan Ahmad, Yasser Bin Salamah

**Affiliations:** Department of Electrical Engineering, College of Engineering, King Saud University, Riyadh 11421, Saudi Arabia; irfahmad@ksu.edu.sa (I.A.); ybinsalamah@ksu.edu.sa (Y.B.S.)

**Keywords:** piezoelectric actuator, nanopositioning, Preisach model, hysteresis, 2-DOF H-infinity control

## Abstract

The nonlinear hysteresis phenomenon can occur in piezoelectric-driven nanopositioning systems and can lead to reduced positioning accuracy or result in a serious deterioration of motion control. The Preisach method is widely used for hysteresis modeling; however, for the modeling of rate-dependent hysteresis, where the output displacement of the piezoelectric actuator depends on the amplitude and frequency of the input reference signal, the desired accuracy cannot be achieved with the classical Preisach method. In this paper, the Preisach model is improved using least-squares support vector machines (LSSVMs) to deal with the rate-dependent properties. The control part is then designed and consists of an inverse Preisach model to compensate for the hysteresis nonlinearity and a two-degree-of-freedom (2-DOF) H-infinity feedback controller to enhance the overall tracking performance with robustness. The main idea of the proposed 2-DOF H-infinity feedback controller is to find two optimal controllers that properly shape the closed-loop sensitivity functions by imposing some templates in terms of weighting functions in order to achieve the desired tracking performance with robustness. The achieved results with the suggested control strategy show that both hysteresis modeling accuracy and tracking performance are significantly improved with average root-mean-square error (RMSE) values of 0.0107 μm and 0.0212 μm, respectively. In addition, the suggested methodology can achieve better performance than comparative methods in terms of generalization and precision.

## 1. Introduction

Piezoelectric actuators (PEAs) are widely used in nanopositioning stages as they can achieve fast and accurate positioning compared with conventional actuators. The main drawback of piezoelectric actuators is that they contain smart materials that exhibit hysteresis behavior, degrading their performance and making them unsuitable for applications that require ultra-precise motion. The reason for this behavior is the change in polarization directions that occurs inside the smart material. This change causes distortion in strain, which is called inhomogeneous domain switching [1,2]. Hysteresis is usually observed as a complex nonlinear relationship between the input (reference signal) and the output (displacement). Therefore, it is difficult to describe the piezoelectric actuator’s dynamics, making the controller design tasks for the nanopositioning systems more difficult [3].

In addition, the hysteresis loops get larger when the input signal contains different frequencies, which also makes the modeling tasks more difficult. This hysteresis type is typically called rate-dependent hysteresis. In this case, the domain-switching process necessitates a specific time duration, affecting the response of piezoelectric materials [4]. The authors in [5] proposed a model to study the impact of the domain structure and the different frequencies of the excitation signals on the shape of hysteresis loops. The results show that the electric field is highly affected by the loading frequencies while slightly affected by the domain shape. The authors in [6] developed a hysteresis model in which the domain-switching process is represented using the constitutive theory. In 2001, finite element techniques were proposed as a means of stimulating microscale models [7]. However, applying this method consumes more execution time.

Among the initial practical endeavors aimed at modeling hysteresis in nanopositioning systems, several simple models, such as the Bouc–Wen model [8], Preisach model [9], Duhem model [10], Dahl model [11], etc., are used. Although these models are, so far, the most popular in hysteresis research, they cannot accurately describe rate-dependent hysteresis. Some of these models have been improved to consider rate-dependent hysteresis, such as the improved Preisach model [12,13] and the improved Prandtl–Ishlinskii model [14,15]. The authors in [16] employed the voltage change rate of the reference signals as inputs to the Preisach model and employed the dynamic mirror coefficient to derive the discretization form. The authors in [17] improved the Bouc–Wen hysteresis model and described the rate-dependent characteristics within the frequency range of 1–50 Hz. The improvement was accomplished by introducing bias parameters to the model. The authors in [18] highlighted the utilization of rate-related factors in the PI model to describe the rate-dependent hysteresis characteristics. However, the output displacements obtained from these models do not align well with the actual displacements. Moreover, the identification of certain unknown parameters within these models can prove to be quite challenging, which in turn makes it difficult to design a control scheme that can successfully achieve high-accuracy compensation. Thus, various methods have been proposed in an attempt to obtain an effective control scheme. For instance, the authors in [19] proposed adaptive feedforward controllers that employ the inverse model of the improved PI model. The authors in [20] developed a modified hysteresis model based on the classical PI model, which includes a quadratic polynomial to describe rate-independent hysteresis, and parameter identification was achieved using self-adaptive particle swarm optimization. However, these control schemes lack robustness in the presence of unmodeled dynamics.

Recently, intelligent models based on machine learning methods, such as artificial neural networks (ANNs) [21] and least-squares support vector machines (LSSVMs) [22], have been put forward to overcome the complexity of the modified classical hysteresis models. These models have attracted attention and gained more popularity than traditional hysteresis models because they can better describe the rate-dependent hysteresis of the PEAs. In addition, these models addressed the nonlinear mapping problem by transforming the multivalued hysteresis mapping into single-to-single mapping. They have also shown great improvements in the generalization ability of models on data with rate-independent and rate-dependent hysteresis. However, the use of the inverse of these models for designing feedforward hysteresis compensators does have some limitations. For instance, some models fall into local optimums and lead to reduced search accuracy [23,24,25].

To overcome the overfitting problem, the authors in [26,27,28,29] proposed hysteresis models based on a regression algorithm. The input of the hysteresis model was selected based on an autoregressive model with exogenous input (NARX), where the current output is dependent not only on the current inputs but also on the past inputs and past outputs. The experimental results indicated that the outputs could be predicted, but the output of the feedforward compensator accumulates errors over time in real-time control experiments due to the feedback [21,30]. In previous studies [31,32], the authors modified the Preisach model using a kernel-based machine learning method combined with a hysteresis memory to overcome the error accumulation limitation. The compensator has been constructed using two parts; hysteresis operators to solve the mapping problem and an inverse Preisach hysteresis model based on the LSSVM algorithm optimized by particle swarm optimization (PSO) and the improved PSO algorithms to estimate the density function. The results showed that the modified Preisach model outperformed the LSSVM-NARX and ANN hysteresis models. However, we used a proportional–integral–derivative (PID) feedback controller to reduce remaining errors, as PID parameter tuning is a time-consuming task and significantly depends on a trial-and-error method through experiments. This makes parameter tuning very difficult to obtain the required performance. Furthermore, PID control is incapable of meeting the robustness and disturbance rejection requirements. Therefore, an alternative feedback controller is needed to avoid the limitations of the classical PID controller and improve positioning accuracy.

In this paper, we developed a robust control strategy to improve the position-tracking performance of a piezoelectric nanopositioning system in the presence of disturbances. A machine-learning method is used to design a feedforward hysteresis compensator. The feedforward controller is then combined with a 2-degree-of-freedom (2-DOF) H-infinity feedback controller for achieving the desired closed-loop tracking performance with robustness and disturbance rejection. This was accomplished by shaping the closed-loop system’s sensitivity functions, with some constraints imposed on these functions to achieve the desired performance. The H-infinity controller has been successfully used to solve the problems of control in many applications [33,34,35,36]. In the H-infinity design, we used a control scheme consisting of two parts: the first one is used in the feedback control for disturbance attenuation and the second one is placed in the feedforward path as a pre-filter to help the Preisach model-based controller in reducing the remaining tracking error. As far as we know from the literature, this hybrid combination has never been employed to control a piezoelectric actuator in nanopositioning systems. To evaluate the effectiveness of our suggested approach, a comparison of the results was made with those obtained from the combination of the Preisach model-based controller with the PID controller, as well as some other related studies.

The remainder of this paper is organized as follows. Details of the experimental setup are presented in Section 2. Hysteresis modeling and system identification are presented in Section 3. A detailed discussion of the feedforward and feedback control strategy is presented in Section 4. Tracking results are presented in Section 5. A comparison with other relevant works is presented in Section 6. Finally, a conclusion is drawn in Section 7.

## 2. Experimental Setup

**Figure 1:**
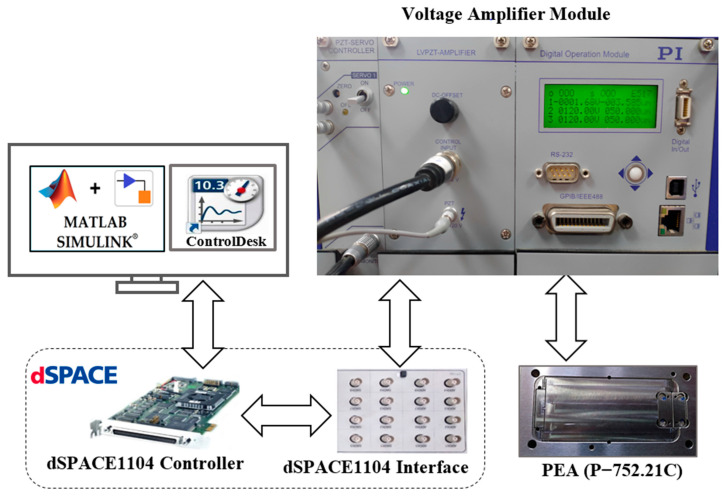
Schematic diagram of the experimental setup for the considered nanopositioning system.

The experimental setup is shown in Figure 1. It consists of a piezo-actuated nanopositioner connected to an amplifier and a PC equipped with a controller card. The nanopositioner (PI GmbH & Co., Karlsruhe, Germany, P-752.21C) [37] is equipped with a flexure hinge mechanism guided by a multilayer piezoelectric ceramic stack actuator. The piezoelectric ceramic material could be induced by an operating voltage of −20 to 120 V to generate deformations, such as expansion and contraction. Thus, this material can force the flexures to move and achieve fast and precise displacement with a motion range of up to 35 µm. The piezo-actuated nanopositioning stage is integrated with a capacitive sensor (PI GmbH & Co., D-015) to measure the displacement. This sensor has a high bandwidth of 10 kHz and can provide a subnanometer resolution of 0.01 nm. The voltage amplifier (PI GmbH & Co., E-505.00) [38] can amplify the input voltage of −2 V to +12 V by a voltage gain factor of 10 so that it can drive the PEA. The control board (dSPACE Co., Wixom, MI, USA, dSPACE 1104) [39] is used to generate the control signals and send or receive the commands through its DAC/ADC ports. First, the control block diagrams are developed using Simulink software. Then, the C code of the developed Simulink model is generated by a compiler supported by dSPACE and connected with the controller using the Real-Time Interface (RTI) library to run the process of the hardware-in-the-loop simulation. For performance evaluation, monitor software (ControlDesk) is used to visualize and save the results.

Different and adequate experimental data representing the rate-independent and rate-dependent hysteresis were collected using this platform. These data (input and output signals of the PEA) were used to train and test both the hysteresis model and the controller. The description of the experimental data will be presented and discussed later. The proposed method used to model and control the PEA is presented in Section 3 and Section 4, respectively.

## 3. Modeling and System Identification

### 3.1. Linear Dynamic Model

**Figure 2:**
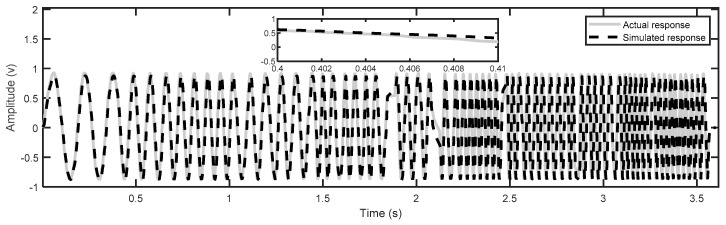
Comparison between the response of the identified model and the response of the experiment.

**Figure 3:**
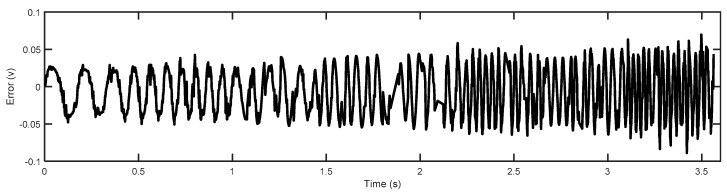
The error between the actual output and the simulated output.

**Figure 4:**
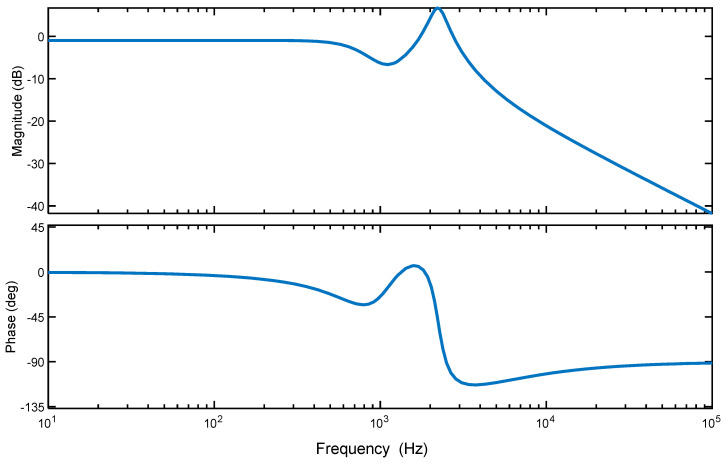
Frequency response of the identified linear dynamic model.

Before modeling the hysteresis, the identification of linear dynamics was performed to find a linear model of the overall system. For this purpose, the excitation of the experimental platform was performed by a sine wave chirp signal with multiple frequencies and an amplitude of 1 V to avoid the appearance of hysteresis in the response. The obtained experimental data were used to characterize the dynamic input–output behavior of the system and identify the plant transfer function. The coefficients of the linear dynamic model were estimated using the recursive least-squares (RLS) method [40]. The transfer function was obtained as follows:

(1) $$G\left(s\right)=\frac{A\left(s\right)}{B\left(s\right)}=\frac{5095s^{3}+1.2\times 10^{8}\;s^{2}+7\times 10^{11}s+4.65\times 10^{15}}{s^{4}+9501s^{3}+2.44\times 10^{8}s^{2}+1.3\times 10^{12}s+5.2\times 10^{15}}$$

where the order of the model dynamics was chosen to be four as it properly describes the system dynamics and matches the actual response well, as shown in Figure 2. Additionally, Figure 3 shows that most error values of the linear dynamic model are within the range of about ±0.05 V. Higher-order models are required to accurately capture the dynamics, but they lead to an increase in the order of the feedback controller, which increases the execution time of the proposed control scheme, thus affecting the applicability of the control design. Figure 4 shows the frequency responses obtained by the identified model; it can be noted that the first resonant mode occurs around 2200 Hz.

### 3.2. Hysteresis Modeling Using a Modified Preisach Model

**Figure 5:**
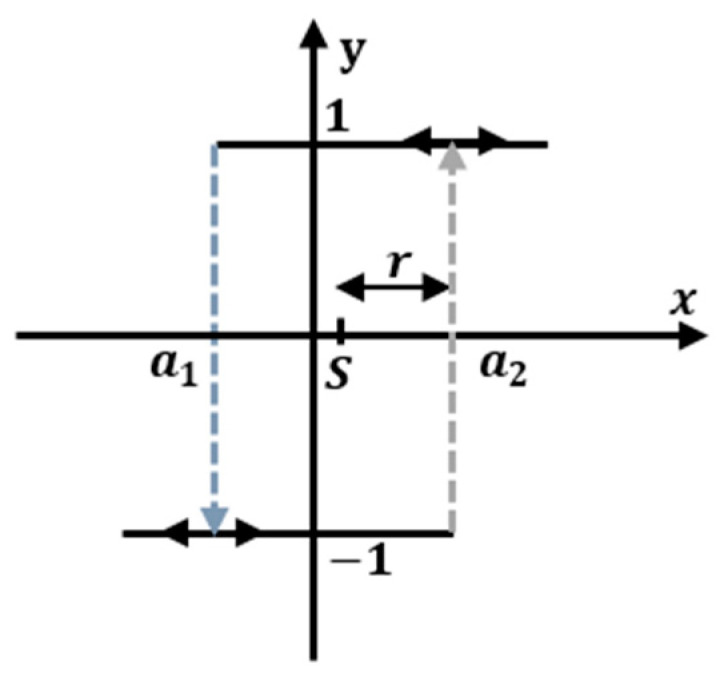
Relay hysteresis operator.

The classical Preisach hysteresis model [41,42] is developed by the relay-type operator $R_{s-r,s+r}\left[\cdot \right]$, as depicted in Figure 5. The relay operator can be expressed as follows:

(2) $$R_{s-r,s+r}\left[x\left(t\right)\right]\{\begin{array}{c} \;1\;\;\;\;\;\;\;\;\;\;\;\;\;\;x\left(t\right)>s+r\;\;\;\;\;\;\;\\ -1\;\;\;\;\;\;\;\;\;\;\;\;\;x\left(t\right)<s-r\;\;\;\;\;\;\\ R_{s-r,s+r}\left[x\left(t-1\right)\right]\;\;\;\;s-r<x\left(t\right)<s+r \end{array}$$

where it is characterized by a couple of swathing thresholds, an upper threshold ($a_{2}=s+r$) and lower threshold ($a_{1}=s-r$), and two states ±1. The Preisach hysteresis model is represented by a continuous linear weighted superposition of these operators, and its output response can be expressed as follows:

(3) $$y\left(t\right)=\overset{\;}{\underset{s+r>s-r}{\iint }}\mu \left(r,s\right)R_{s+r,s-r}\left[z\right]\left(t\right)dsdr$$

where μ(r,s) denotes the weight function (or density function) and $R_{s+r,s-r}$ denotes the basic hysteretic unit (hysteron) in the Preisach plane *P* = $\left\{\left(s+r,s-r\right):\;s+r\geq s-r,\;s+r<a_{2},\;s-r<a_{1}\right\}$.

**Figure 6:**
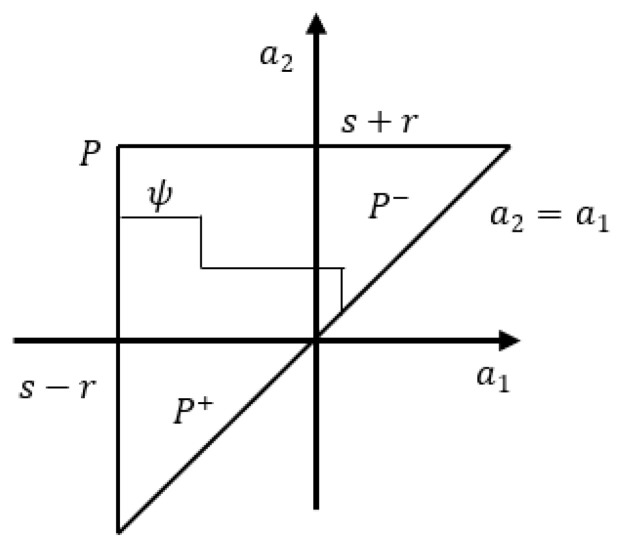
Preisach model.

Suppose that ψ is a curve that divides the Preisach plane *P* into two different areas; a positive area $P^{+}$, in which the relays take the output values of +1, and a negative area $P^{-}$, in which the relays take the output values of −1, as depicted in Figure 6. In this case, the output response of the Preisach model can be rewritten as:

(4) $$y\left(t\right)=\int_{0}^{+\infty }\left[\int_{-\infty }^{\psi \left(t,r\right)}\mu \left(r,s\right)ds-\int_{\psi \left(t,r\right)}^{+\infty }\mu \left(r,s\right)ds\right]dr$$

or:

(5) y(t)=Q(r, ψ(t,r))

In Equations (4) and (5), it can be seen that the Preisach hysteresis model is composed of a set of continuous hysteresis operators and a density function. The key issue with the classical Preisach model is finding a high-accuracy approximation method for the density function. Furthermore, this algorithm should achieve reasonable execution times, in the sense that the time complexity should be taken into account at the various stages of the modeling and control development process.

**Figure 7:**
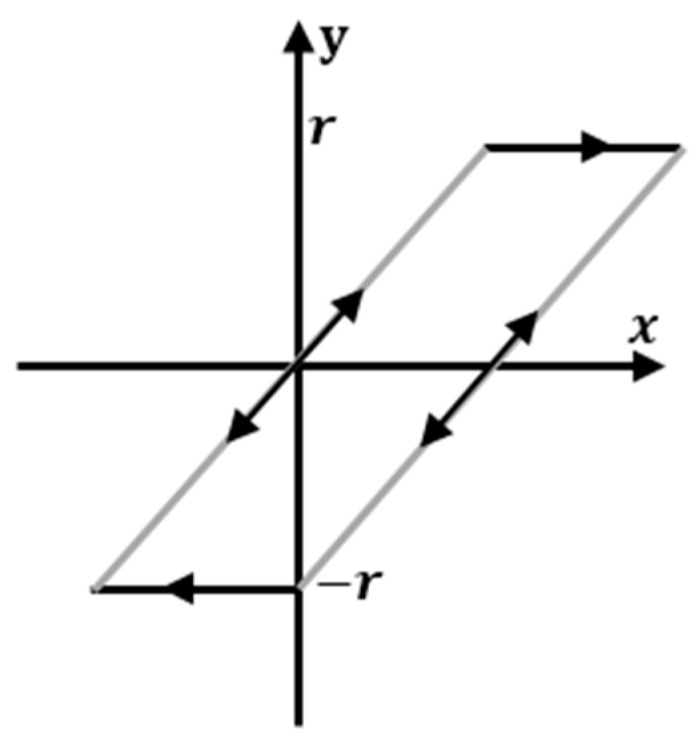
Stop hysteresis operator.

In this study, the Preisach plan was divided into a set of intervals (cells) to reduce the complexity of the model, and then the density function was approximated by using a regression method to improve the accuracy of identification and enhance the generalization ability of the model. In this case, we used the stop operator, as depicted in Figure 7. This operator is represented by a couple of thresholds, *+r* and *−r,* which can be defined as:

(6) $$r_{i}=\frac{i}{\left(n+1\right){\left|x\right|}_{max}},\;i=1,\;2,\;3,\ldots ,n$$

where n indicates the number of stop operators and ${\left|x\right|}_{max}$ indicates the maximum absolute value of reference signal amplitudes. The output response of this operator on an interval from $t_{i}$ to $t_{i+1}$ can be expressed as:

(7) $$\begin{array}{c} z\left(0\right)=E_{r}\left[x\left(0\right)\right]\\ z\left(t\right)=E_{r}\left[x\left(t\right)-x\left(t_{i}\right)+z\left(t_{i}\right)\right] \end{array}$$

where:

(8) $$E_{r}\left[\cdot \right]=min\left\{max\left\{-r,.\right\},+r\right\}$$

where z(t) denotes the current state of the operator at a certain time and $E_{r}\left[\cdot \right]$ denotes the stop operator.

Then, the outputs of the stop operators were used as inputs to the prediction algorithm. This predictor was built by using the least-squares support vector machine (LSSVM) [22,43]. The LSSVM combines the advantages of replacing inequality constraints with equality constraints and adopting the error sum of the square loss function in the cost function rather than the insensitive loss function of the standard SVM. These important simplifications linearize the problem and make the algorithm a powerful tool for solving regression problems. Basically, the LSSVM regression for input–output data pairs (*z*, *y*) is defined as:

(9) $$y\left(z\right)=w^{T}\emptyset \left(z\right)+b$$

where ∅(·) denotes a nonlinear function that maps the input data *z* into a high-dimensional feature space *y*, w denotes the dimensional weight vector, and *b* denotes mapping bias. In the training phase, the parameters w and *b,* as well as the parameters of ∅(·), should be well determined to yield a high degree of goodness-of-fit between the actual and predicted output displacement of the piezoelectric actuator in the test phase. For this purpose, the optimization problem for the LSSVM is formulated as:

(10) $$\underset{w,e,b}{min}J_{p}\left(w,e\right)=\frac{1}{2}w^{T}w+C\frac{1}{2}\overset{N}{\underset{k=1}{\sum }}e_{k}^{2}$$

$$\mathrm{Subject}\;\mathrm{to}\;y_{k}=w^{T}\emptyset \left(z^{k}\right)+b+e_{k}$$

where $e_{\;}$ denotes the error between the actual and predicted output displacement and *C* denotes the regularization parameter, which determines the balance between the training error minimization and smoothness of the regression function and is directly related to the generalization ability of the model. By using the Lagrangian function, the cost function of Equation (10) can be rewritten as:

(11) $$ℒ\left(w,b,e;\alpha \right)=J_{p}\left(w,e\right)-\overset{N}{\underset{k=1}{\sum }}\alpha_{k}\left[w^{T}\emptyset \left(z^{k}\right)+b+e_{k}-y_{k}\right]$$

where $\alpha_{\;}$ denotes the Lagrange multiplier. The optimal solution for this problem can be obtained by using the Karush–Kuhn–Tucker (KKT) conditions [44]. The KKT conditions are defined by solving the partial derivatives on ℒ(w,b,e;α) with respect to $w,b,e_{k}$ and $\alpha_{k}$ as follows:

(12) $$\frac{\partial L}{\partial w}=0\to w=\overset{N}{\underset{k=1}{\sum }}\alpha_{k}\emptyset \left(z^{k}\right)$$

(13) $$\frac{\partial L}{\partial e_{k}}=0\to \alpha_{k}=Ce_{k}$$

(14) $$\frac{\partial L}{\partial b}=0\to \overset{N}{\underset{k=1}{\sum }}\alpha_{k}=0$$

(15) $$\frac{\partial L}{\partial \alpha_{k}}=0\to w^{T}\emptyset \left(z^{k}\right)+b+e_{k}-y_{k}$$

Thus, the linear equations can be derived after the elimination of w  and $e_{k}$ as follows:

(16) $$\left[\begin{array}{cc} 0 & 1_{N}^{T}\\ 1_{N} & Ω+I/C \end{array}\right]\left[\begin{array}{c} b\\ \alpha \end{array}\right]=\left[\begin{array}{c} 0\\ y \end{array}\right]$$

where $1_{N}$ denotes a unity vector, *I* denotes the identity matrix, and Ω is a matrix that can be calculated by multiplying $\emptyset \left(z^{k}\right)$ with $\emptyset \left(z^{j}\right),$ where {*j, k =* 1,2, …., *N*} and *N* denote the number of samples in the training data. To calculate the matrix Ω using a simplified form, we used a radial base function (RBF) kernel function:

(17) $$K\left(z^{k},z^{j}\right)=\emptyset \left(z^{k}\right)\emptyset \left(z^{j}\right)$$

(18) $$\mathrm{where}\;K\left(z,z^{k}\right)=exp\left(-\frac{\left\|z-z^{k}\right\|}{\sigma^{2}}\right)$$

where σ denotes the kernel parameter, which has to be tuned to find the optimal variance of the Gaussian function. Like the regularization parameter, the kernel parameters are also directly related to the generalization ability of the model. The LSSVM regression model can finally be expressed as:

(19) $$\hat{y}\left(z\right)=\sum_{k=1}^{N}\alpha_{k}K\left(z,z^{k}\right)+b$$

The particle swarm optimization (PSO) technique [45,46] was applied to optimize the hyper-parameters *C* and $\sigma^{\;}$ of the LSSVM model. It is a robust optimization technique for solving optimization search problems. Basically, the PSO algorithm defines the search as:

(20) $$\begin{array}{c} v_{i}\left(t\right)=\eta v_{i}\left(t-1\right)+c_{1}r_{1}\left(p_{best,i}-p_{i}\left(t-1\right)\right)+c_{2}r_{2}\left(g_{best}-p_{i}\left(t-1\right)\right)\\ p_{i}\left(t\right)=p_{i}\left(t-1\right)+v_{i}\left(t\right) \end{array}$$

where $p_{\;}$ and $v_{\;}$ denote the position and the velocity of a particle in iteration *i*, respectively. *η* denotes the inertia weight, which has to be tuned to provide a tradeoff between local search and global search, $g_{best}$ denotes the global optimal position, $p_{best,i}$ denotes the local optimal position, $c_{1}$ and $c_{2}$ are the acceleration parameters, and $r_{1}$ and $r_{2}$ denote random numbers randomly selected between 0 and 1.

**Figure 8:**
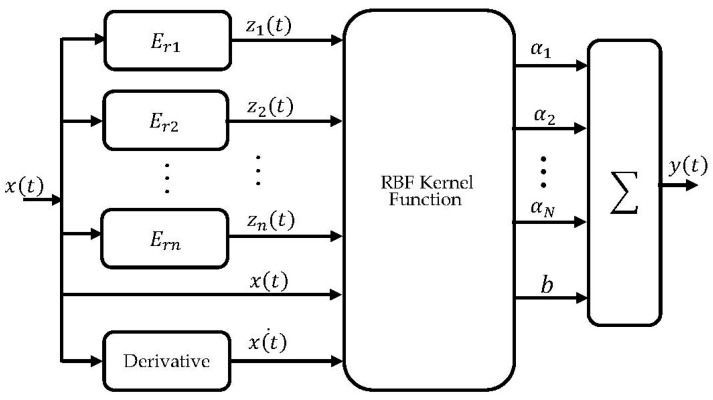
Block diagram of the modified Preisach model.

For a satisfactory generalization ability, we included the derivative of the input reference signals, as it helps to describe the rate-dependent characteristics that are based on both input voltage and input rate. Figure 8 shows how the overall structure of the proposed modified Preisach hysteresis model was built. This model was trained, and the simulation results will be presented and discussed in the next subsection.

### 3.3. Hysteresis Modeling Results

**Figure 9:**
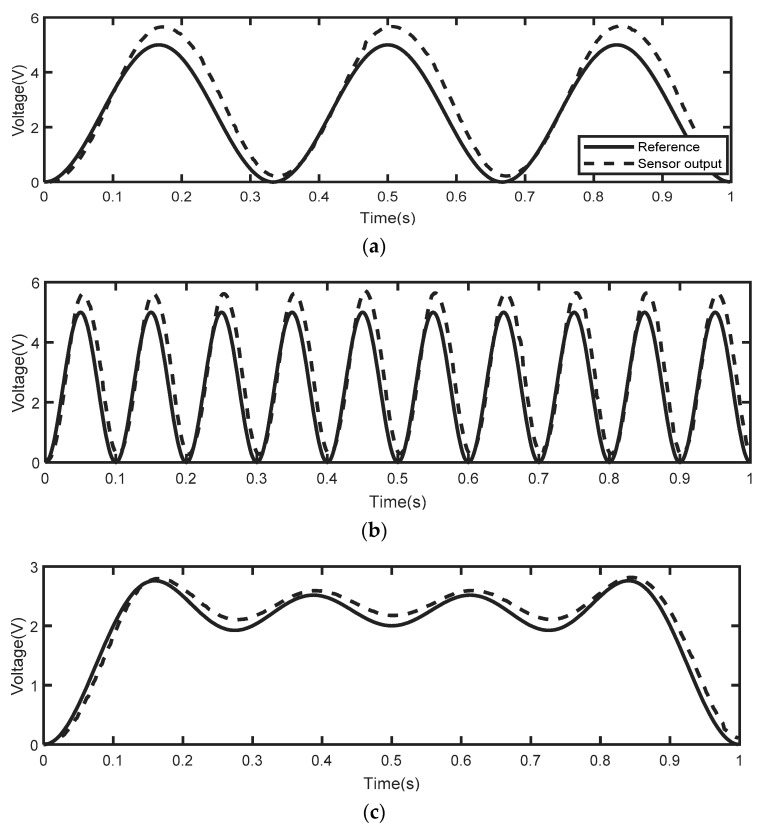
The excitation signals of the PEA in the training phase and the corresponding output signals of the sensor: (**a**) first training signal; (**b**) second training signal; (**c**) third training signal.

The simulation of the dynamic hysteresis model was carried out under the MATLAB-2022b environment (MathWorks, Natick, MA, USA). The PSO-LSSVM algorithm was implemented with the help of the Least-Squares SVM-MATLAB Toolbox 1.8 (KU Leuven, Leuven, Belgium) [47]. The model was trained using datasets that were selected to involve different values of amplitudes and frequencies. These datasets consist of the excitation signals that were sent to the PEA and the corresponding output signals obtained from the sensor, as shown in Figure 9. Each training signal contains 500 samples (with a sample time of 0.02 s), and their amplitudes range from 0 to 6 V before amplification. In our previous work [31], we noted an interesting tradeoff between model complexity and accuracy and proved that the optimal number of operators is 55; thus, we used the same number in this study as well. Additionally, in the same previous paper, we presented a detailed discussion of the optimal tuning of PSO hyper-parameters used for training the model. All parameter values obtained through the Preisach modeling of the dynamic hysteresis behavior are illustrated in Table 1.

**Figure 10:**
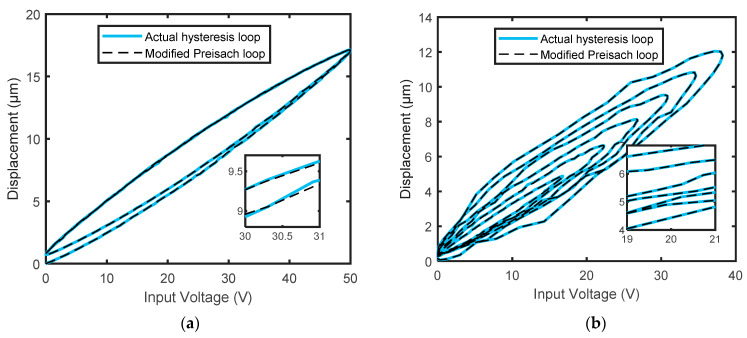
Comparison between the actual hysteresis loops and the predicted hysteresis loops for the considered actuator: (**a**) data A; (**b**) data B.

**Figure 11:**
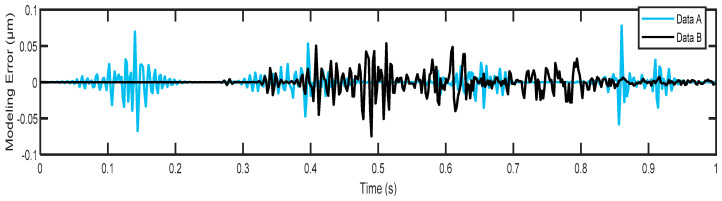
The instantaneous prediction errors obtained from the difference between the actual and predicted responses for test data; A and B.

The effectiveness of the proposed hysteresis model was validated by two test datasets, as shown in Figure 10. This figure shows the actual hysteresis loops against the predicted hysteresis loops for the considered actuator. It can be seen that there is a good match between them, in the sense that the model achieved good predictive performance. The root-mean-square-error (RMSE) was used as a measure of modeling quality. The suggested model achieved low prediction error values in test datasets, A = 0.01061 μm and B = 0.01086 μm, with an average RMSE of 0.0107 μm. For further comparison, we also plotted the instantaneous prediction errors in Figure 11. It can be observed that most error levels are within the range of ±0.05 μm, which indicates that our model yields an extremely good fit, and hence its inverse can be amenably used for designing a practical compensator.

## 4. Hysteresis Compensation

In this section, the proposed control scheme with all the necessary details is presented.

### 4.1. Feedforward–Feedback Control Structure

**Figure 12:**
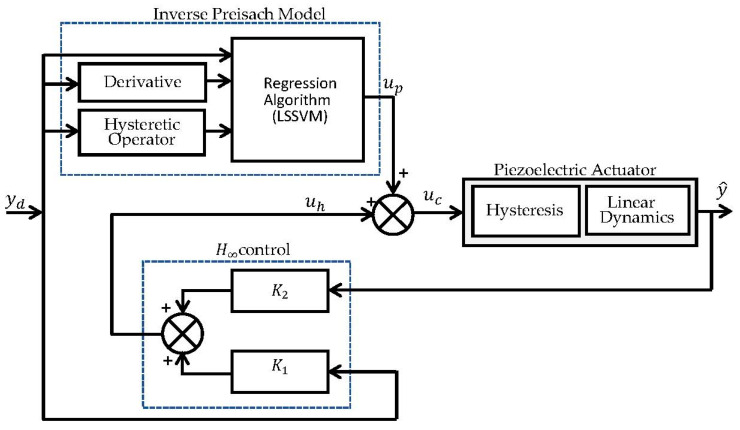
Block diagram of the proposed nanopositioning control scheme.

For hysteresis compensation and precise reference tracking, a compound control scheme consisting of a feedforward (FF) compensation part and a robust feedback control part was used, as shown in Figure 12. This method integrates the modified Preisach inverse model-based controller for hysteresis compensation and a two-degree-of-freedom (2-DOF) controller based on the H-infinity control method to improve the tracking performance with robustness and disturbance rejection. The parameter identification of the inverse hysteresis model was performed by training the modified Preisach model inversely as its input was used as an output, and vice versa. Once the training phase is completed, the inverse Preisach hysteresis model is used to construct the FF control part. The inverse model compensator is widely used in nanopositioning applications; however, it has a disadvantage as its tracking performance relies significantly on the accuracy of the inverse hysteresis model employed in the design. Thus, eliminating the overall effect of hysteresis by only placing its approximate inverse model in a feed-forward path is often challenging [48,49].

Therefore, we combined the FF control part with a feedback control method to reduce any remaining tracking errors. There would be significant advantages to inserting robust control in the feedback path to eliminate modeling errors and handle any external disturbances. For this purpose, a 2-DOF robust H-infinity control [50] was used in this study. The H-infinity theory is widely used for developing robust control methods to maintain local stability and achieve satisfactory performance. The considered 2-DOF robust H-infinity feedback controller consists of two parts; the first part ($K_{1}$) is placed in the feedforward path as a pre-filter to help the Preisach model-based controller in reducing the remaining tracking error, whereas the second part ($K_{2}$) is used in the feedback path for any possible disturbance rejection. Details of the H-infinity control design used in this paper will be further discussed in the next subsection.

### 4.2. Two-Degree-of-Freedom (2-DOF) H-Infinity Controller Design

**Figure 13:**
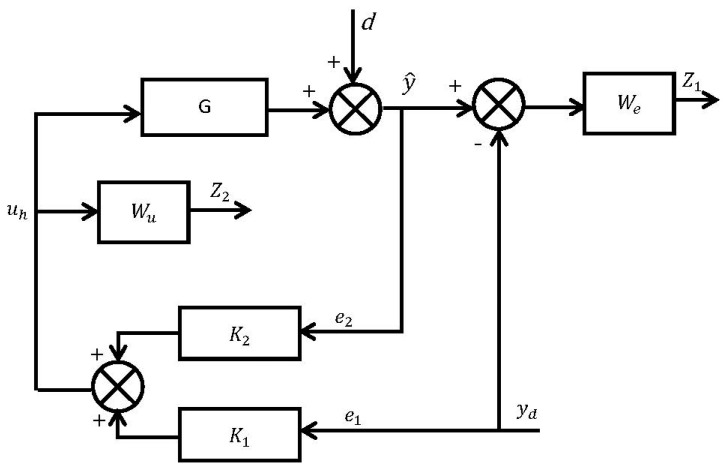
Block diagram for the proposed 2-DOF $H_{\infty }$ controller design.

The feedback controller should be designed so that the closed-loop system is robust and can achieve the desired tracking performance. For this purpose, the two-degree-of-freedom $H_{\infty }$ optimization method [50] was adopted in our study. The scheme of this control strategy is shown in Figure 13. The system has a reference signal ($y_{d}$) and external disturbance (*d*). *G* is the nominal plant model of the considered system, given by Equation (1), which is used for the synthesis of the $H_{\infty }$ controller, and $K_{1}$ and $K_{2}$ are the $H_{\infty }$ controllers to be designed. Therefore, the output response, control signal, and error signals of the closed-loop systems can be expressed as follows:

(21) $$\hat{y}=\frac{K_{1}G}{1-GK_{2}}y_{d}+\frac{1}{1-GK_{2}}d$$

(22) $$u_{h}=\frac{K_{1}}{1-GK_{2}}y_{d}+\frac{K_{2}}{1-GK_{2}}d$$

(23) $$e_{1}=y_{d}$$

(24) $$e_{2}=\frac{K_{1}G}{1-GK_{2}}y_{d}+\frac{1}{1-GK_{2}}d$$

In the above equations, it is clear that we should minimize the sensitivity function ${\left(1-GK_{2}\right)}^{-1}$ for good disturbance attenuation and $K_{1}{\left(1-GK_{2}\right)}^{-1}$ and $K_{2}{\left(1-GK_{2}\right)}^{-1}$ for less control energy to avoid saturation issues. Similarly, for the frequency range of the reference input trajectory, the sensitivity function $K_{1}G{\left(1-GK_{2}\right)}^{-1}$ must be at 0 dB in order to achieve precise reference tracking. This can be performed by imposing some templates in terms of weighting functions on the shapes of the closed-loop sensitivity functions.

**Figure 14:**
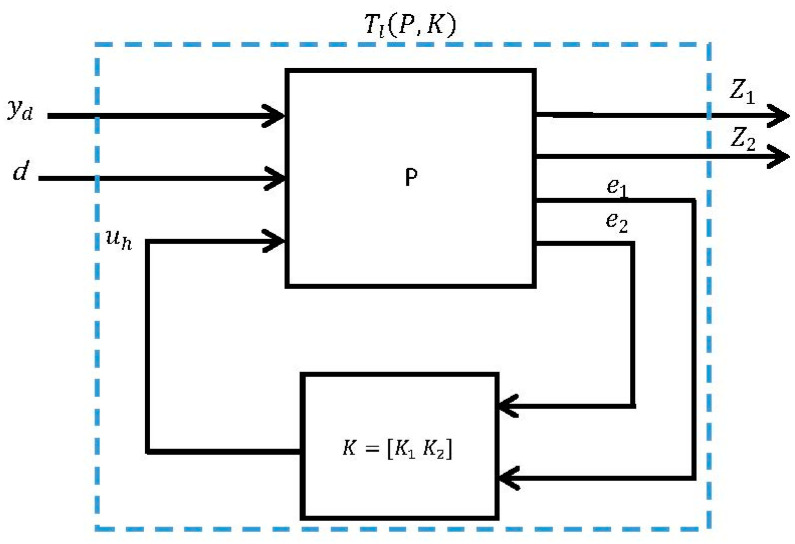
Generalized block diagram with a 2-DOF $H_{\infty }$ controller design.

Two weighting functions were suggested for $H_{\infty }$ controller design. $W_{u}$ is a weighting function on the control signal and $W_{e}$ is a weighting function on the error signal ($\hat{y}-y_{d}$). The closed-loop system was then represented in the standard configuration using the lower linear fractional transformation (LLFT) technique [51], as shown in Figure 14. Here, P represents the generalized plant model and $z_{1}$ and $z_{2}$ are the two controlled outputs. More details about this method are available in [50]. The system is, therefore, described as follows:

(25) $$\left[\begin{array}{c} z_{1}\\ z_{2}\\ e_{1}\\ e_{2} \end{array}\right]=\left[\begin{array}{ccc} -W_{e} & W_{e} & W_{e}G\\ 0 & 0 & W_{u}\\ 1 & 0 & 0\\ 0 & 1 & G \end{array}\right]\left[\begin{array}{c} y_{d}\\ d\\ u_{h} \end{array}\right]$$

and the closed-loop transfer function matrix $T_{l}\left(P,\;K\right)$ is given by:

(26) $$T_{l}\left(P,\;K\right)=\left[\begin{array}{cc} W_{e}\left(S_{O}GK_{1}-1\right) & W_{e}S_{O}\\ W_{u}S_{i}K_{1} & W_{u}K_{2}S_{O} \end{array}\right]$$

where $S_{i}={\left(1-K_{2}G\right)}^{-1}$ is the input sensitivity function and $S_{o}={\left(1-GK_{2}\right)}^{-1}$ is the output sensitivity function. Therefore, in this case, the goal is to minimize the $H_{\infty }$ norm of $T_{l}\left(P,\;K\right)$. A description of each element of $T_{l}\left(P,\;K\right)$ is given in Table 2.

Hence, the generalized optimization problem of the $H_{\infty }$ controller design is given as follows:

(27) $$min{\left\|T_{l}\left(P,\;K\right)\right\|}_{\infty }=min\left[\underset{\omega }{max}\bar{\sigma }\;\left(T_{l}\left(P,\;K\right)\left(j\omega \right)\right)\right]$$

where $\underset{\;}{max}\bar{\sigma }\left(\cdot \right)$ denotes the maximum singular value of $T_{l}\left(j\omega \right)$.

### 4.3. Performance Specifications and Robustness Analysis

We used the $H_{\infty }\;$ suboptimal problem so that the $H_{\infty }$ norm of the closed-loop transfer function is less than a specified positive value (γ=1), as given below:

(28) $${\left\|T_{l}\left(P,\;K\right)\right\|}_{\infty }<1$$

The closed-loop sensitivity functions must remain under the magnitudes of the inverse of corresponding weighting functions, where:

$$\left|S_{O}GK_{1}-1\right|<\frac{1}{W_{e}},\;\left|S_{O}\right|<\frac{1}{W_{e}},\;\left|S_{i}K_{1}\right|<\frac{1}{W_{u}},\;\left|K_{2}S_{O}\right|<\frac{1}{W_{u}}$$

The constraints imposed on the sensitivity functions method are demonstrated in Table 3.

In order to achieve the above-mentioned constraints, the weighting functions were chosen as follows:

(29) $$W_{e}\left(s\right)=\frac{0.505s+248.4}{s+17.59}$$

(30) $$W_{u}\left(s\right)=\frac{50s+3936}{s+42,800}\;\;$$

**Figure 15:**
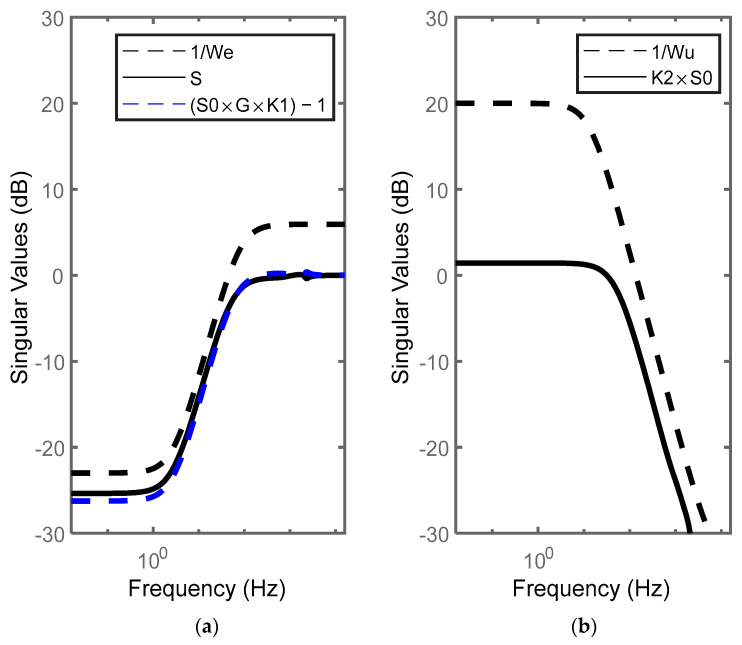
Comparison of the desired response of the sensitivity functions with the response of the inverse weighting functions: (**a**) output sensitivity function response $S_{O}$ and $\left(S_{O}GK_{1}-1\right)$ with $1/W_{e}$; (**b**) $K_{2}S_{O}$ with $1/W_{u}$.

**Figure 16:**
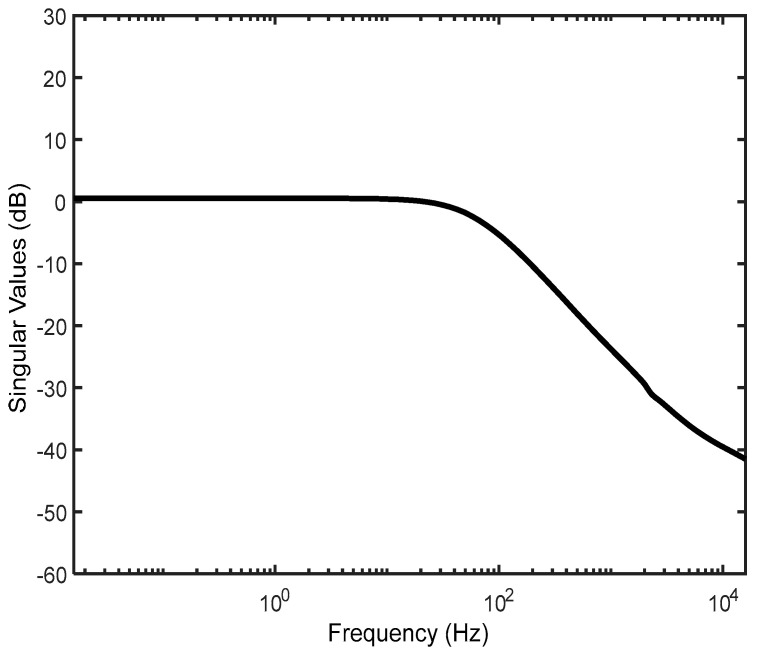
Complementary sensitivity function.

Thus, the transfer functions of the optimal controllers $K_{1}$ and $K_{2}$ were obtained as follows:

(31) $$K_{1}\left(s\right)=\frac{K_{n}\left(s\right)}{K_{d}\left(s\right)}=\frac{n_{1}s^{6}+n_{2}s^{5}+n_{3}s^{4}+n_{4}s^{3}+n_{5}s^{2}+n_{6}s+n_{7}}{s^{7}+d_{1}s^{6}+d_{2}s^{5}+d_{3}s^{4}+d_{4}s^{3}+d_{5}s^{2}+d_{6}s+d_{7}}$$

(32) $$K_{2}\left(s\right)=\frac{K_{p}\left(s\right)}{K_{z}\left(s\right)}=\frac{p_{1}s^{6}+p_{2}s^{5}+p_{3}s^{4}+p_{4}s^{3}+p_{5}s^{2}+p_{6}s+p_{7}}{s^{7}+z_{1}s^{6}+z_{2}s^{5}+z_{3}s^{4}+z_{4}s^{3}+z_{5}s^{2}+z_{6}s+z_{7}}$$

where the parameters of these controllers are given in Table 4. The designed controller meets the desired performance objectives and constraints with a minimal achievable value ($min({\left\|T_{l}\left(P,K\right)\right\|}_{\infty })$) of 0.984. The sensitivity functions lie under the curves of the constraints represented by $1/W_{e}$ and $1/W_{u}$, as shown in Figure 15 and Figure 16. These figures show good modulus margins, $\|S_{0}\|{}_{\infty }=0.077$ dB and $\|K_{2}S_{O}\|{}_{\infty }=1.427$ dB, and a satisfactory tracking bandwidth that covers the reference signals used in this study. These results indicate that the required good performance and disturbance rejection requirements are fully met with our control design.

## 5. Tracking Results

**Figure 17:**
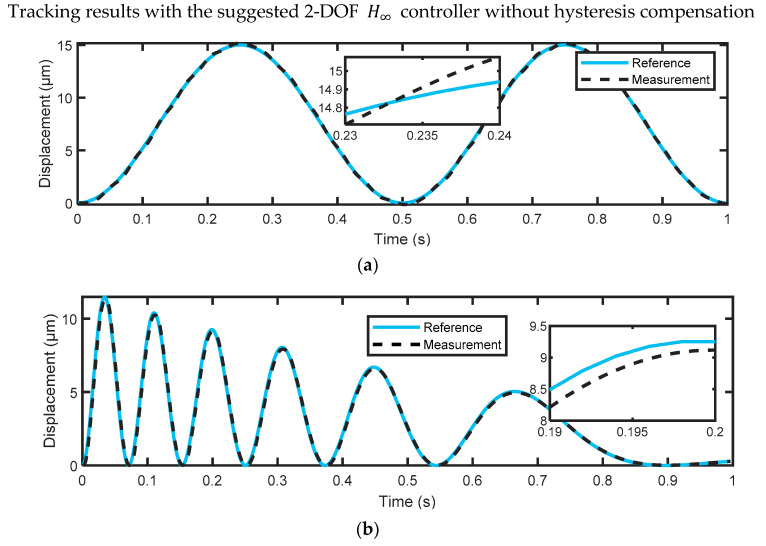
Tracking results obtained by the 2-DOF $H_{\infty }$ controller without hysteresis compensation for (**a**) reference signal A; (**b**) reference signal B.

**Figure 18:**
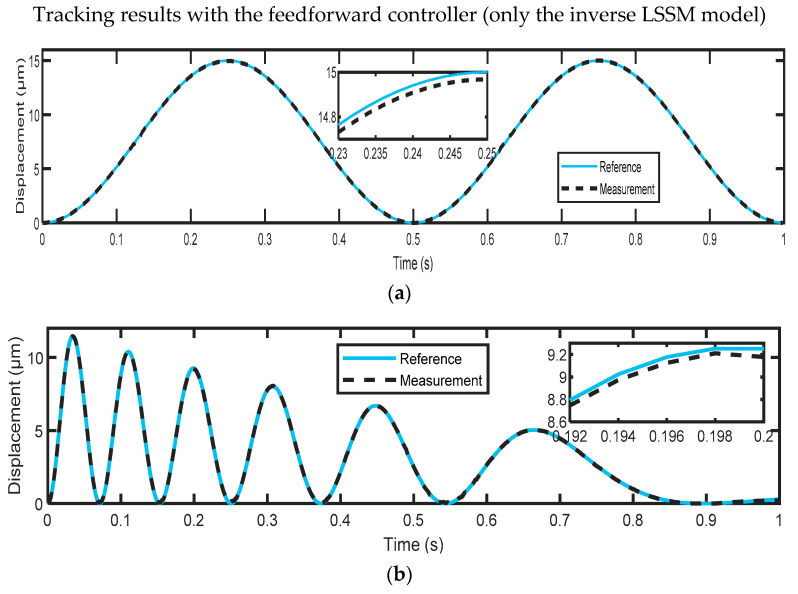
Tracking results obtained by only the inverse LSSVM model-based controller for (**a**) reference signal A; (**b**) reference signal B.

For the evaluation of the proposed control scheme, the test reference signals mentioned in Section 3.3 were used to excite the considered PEA and then compared with the corresponding measured output displacements. For better performance evaluation, the root-mean-square error (RMSE) was used to measure the tracking error, which is suitable for the measurement of tracking errors for nanopositioning systems. Figure 17 shows the tracking results with only the 2-DOF $H_{\infty }$ feedback controller without the hysteresis feedforward compensator for two test datasets. As the $H_{\infty }$ feedback controller was designed for a linear plant model and the nonlinear hysteresis of the actuator was not compensated for in this case, large tracking errors (A = 0.1006 μm and B = 0.1664 μm) were observed, as expected. As depicted in Figure 18, the tracking error has been significantly reduced to A = 0.0258 μm and B = 0.0361 μm when utilizing only the hysteresis feedforward compensator, resulting in improved tracking performance.

**Figure 19:**
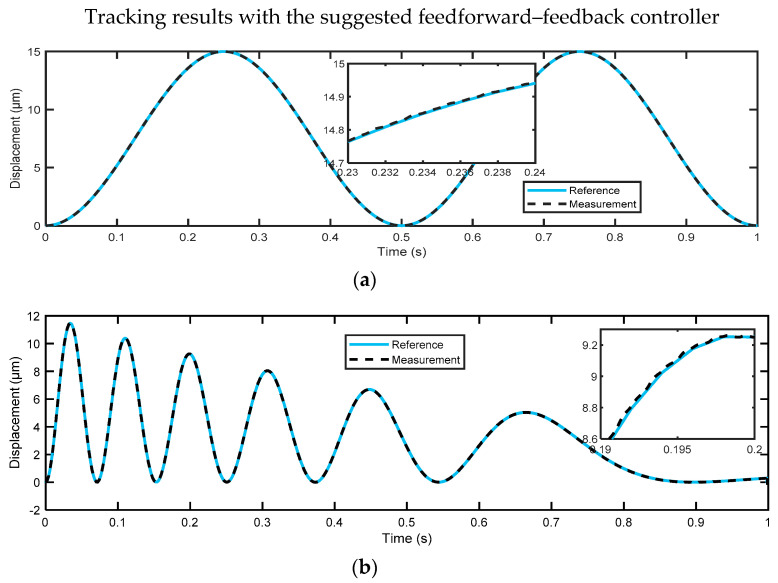
Tracking results obtained by the proposed control scheme for (**a**) reference signal A; (**b**) reference signal B.

**Figure 20:**
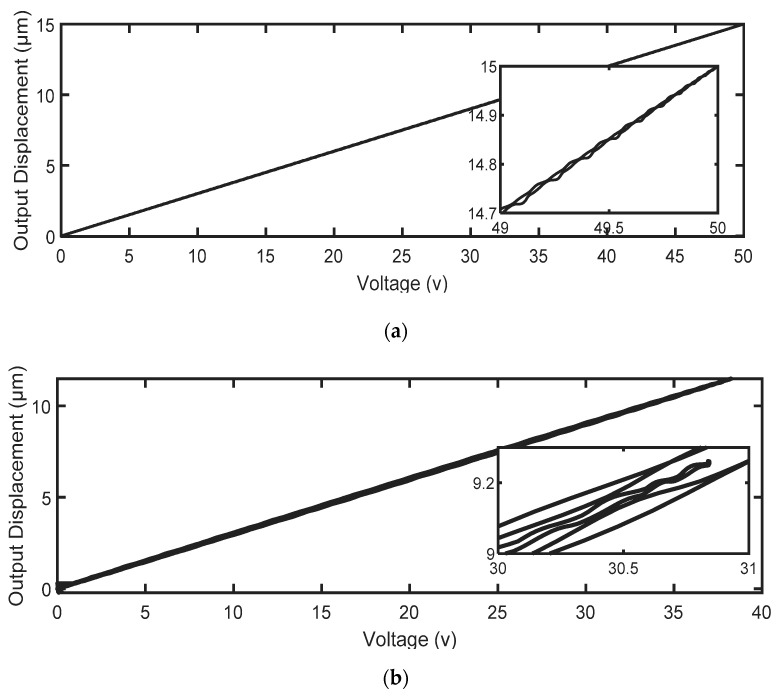
The input–output relationship obtained by the proposed control for (**a**) reference signal A; (**b**) reference signal B.

Further improvements in the tracking performance have been achieved by utilizing the 2-DOF $H_{\infty }$ feedback controller in the presence of the nonlinear hysteresis feedforward compensator, as illustrated in Figure 19. The proposed control design effectively compelled the system’s output to closely follow the reference trajectory, achieving an RMSE of 0.019 μm and 0.0233 μm, respectively. These results indicate that the feedforward hysteresis compensator does not have an adverse effect in the presence of the 2-DOF $H_{\infty }$ feedback controller, which has two parts (*K*_1_ and *K*_2_). Nonlinear hysteresis compensation with an inverse hysteresis feedforward compensator is indeed required with a feedback controller, which is designed for a linear plant model in order to achieve the desired tracking performance. Figure 20 shows the effectiveness of our controller on the considered nanopositioning system in reducing the hysteresis nonlinearity of the PEA, where it describes a highly linear relationship between the input and output.

**Figure 21:**
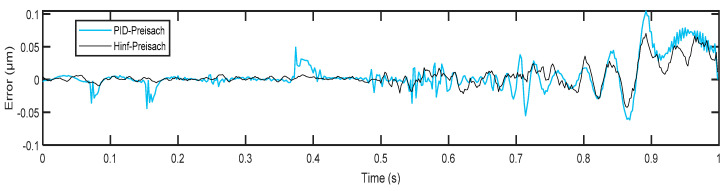
Comparison of the proposed control scheme with the PID–Preisach control scheme in terms of error levels in dataset B.

Table 5 shows the tracking performance for the proposed control scheme compared with the LSSVM-PID feedback controller, which has been proposed in our previous work [31]. It can be observed that the 2-DOF $H_{\infty }$ controller achieved better trajectory tracking performance than the traditional PID feedback controller, obtaining a 0.0212 μm RMSE on average while the PID achieved a 0.0241 μm RMSE on average. To further clarify the difference, the instantaneous tracking errors for both schemes were compared on test dataset B, as shown in Figure 21. These differences, in terms of mean RMSE and error levels, may seem small, but their effect is astonishing in nanoscale systems.

The above results presented in this study were achieved after carefully considering the tradeoff between two critical factors: tracking performance and system robustness. It is well-known that improving tracking performance often requires sacrificing system robustness while enhancing system robustness typically results in decreased tracking performance. Therefore, the tracking error shown in Figure 21 could be further reduced by modifying the template of the weighting function imposed on the closed-loop sensitivity function in the $H_{\infty }\;$ design, but this comes at the cost of lower robustness and stability margins of the system.

## 6. Comparison with Other Relevant Works

In this section, we compare our results to some existing results in related works mentioned in Section 1. The results are shown in Table 6, where we can see that our method improves tracking performance on nanopositioning systems and outperforms in comparison with the other methods in terms of average RMSE. The compensation method that is based only on the improved inverse Preisach [52] method has low accuracy (RMSE of =0.15 μm), but it is better than the compensation method based on the recurrent neural networks (RNNs) [25], which produced an average RMSE of 0.465 μm. The FF-FB control method based on the LSSVM algorithm without modeling hysteresis [28] produced the highest RMSE value (0.62 μm). The authors in [26] have tried to apply the same algorithm (LSSVM) to model hysteresis based on an autoregressive model with exogenous input (NARX), as they reduced the average tracking error to 0.03 μm. In our previous work [31], we designed a hybrid control scheme consisting of an FF controller developed by modified Preisach using the PSO-LSSVM algorithm, whereas the FB controller was developed by incremental PID control, where the average tracking error was reduced to 0.0241 μm for the same test dataset. The performance improvement strategy in this paper has a better average tracking error (0.0212 μm) than the PID-LSSVM controller. In addition, Table 6 also compares our results with those obtained by other studies that used the same piezoelectric actuator (P-752.21C), as our presented control strategy using the 2-DOF $H_{\infty }\;$ robust feedback controller achieved better tracking performance than comparative studies in terms of the RMSE. This comparison demonstrated that our method is more powerful and has a higher degree of generalization than the other methods in handling the effects of the nonlinearities of the PEA on nanopositioning systems.

## 7. Conclusions

This paper presents a new compound control architecture in such a way that it can handle nonlinearities and enhance the tracking performance of the control system for a piezoelectric actuator. The author created a robust controller by combining the improved inverse Preisach hysteresis model with the 2-DOF $H_{\infty }$ control. The PSO-LSSVM algorithm and the hysteresis stop operator algorithm were used to model the hysteresis response and design the inverse of the Preisach hysteresis. For H-infinity control, the two-degree-of-freedom H-infinity control strategy was used to provide robust performance under external disturbances.

The achieved results show that the hysteresis model developed in this study can yield accurate results, with an average prediction accuracy of 0.0107 μm. In addition, it has been found that the feedforward controller, which uses only the inverse LSSM model, achieved better tracking results compared to the suggested 2-DOF $H_{\infty }$ controller without hysteresis compensation. For dataset A, the feedforward controller had a tracking error of 0.0258 μm, while the $H_{\infty }$ controller had a tracking error of 0.1006 μm. Similarly, for dataset B, the feedforward controller had a tracking error of 0.0361 μm, while the $H_{\infty }$ controller had a tracking error of 0.1664 μm. The combination of both control approaches produced the best tracking results, with a tracking error of 0.019 μm for dataset A and 0.0233 μm for dataset B. This approach was superior to some of the recent approaches described in the literature, which means that our control scheme is highly capable of dealing with disturbances and compensating for PEA nonlinearities, as it could precisely track the reference input signals with an average tracking precision of 0.0212 μm.

It is possible to further reduce the tracking error obtained by the proposed control method by modifying the template of the weighting function imposed on the closed-loop sensitivity function in the $H_{\infty }$ design. However, doing so comes at the cost of lower robustness and stability margins of the system. Additionally, increasing the number of hysteresis operators can also improve the results, but it comes with a tradeoff of increased computational complexity of the control part, which can affect the applicability of the control system.

## Figures and Tables

**Table 1:** The parameter values obtained for the Preisach hysteresis model.

Parameter	Value
Samples/second	500
Range of reference signal amplitudes	0–6 V
Number of hysteresis operators	55
Acceleration parameters	2
Population size	30
Minimum and maximum inertia weights of particles	[0.4, 0.9]
Iterations	100
Hyper-parameters	*C* = $3.81\times 10^{3}$, $\sigma^{2}$ = 3.135
Bias	1.831

**Table 2:** Description of H-infinity functions that have to be minimized for the proposed control design.

Function	Description
$W_{e}\left(S_{O}GK_{1}-1\right)$	The weighted error between the ideal and actual closed-loop system
$W_{e}S_{O}$	Weighted output sensitivity
$W_{u}S_{i}K_{1}$	Weighted control action that can occur due to reference
$W_{u}K_{2}S_{O}$	Weighted control action that can occur due to disturbance

**Table 3:** The constraints imposed on the sensitivity functions.

Constraint	Description
${\left\|S_{0}\right\|}_{\infty }<6\;\mathrm{dB}$	For good robustness and sufficient stability margin
$\left|S_{0}\right|<-23\;\mathrm{dB}$	For good disturbance attenuation and less tracking error
$\left|S_{i}K_{1}\right|<20\;\mathrm{dB}$ $\left|K_{2}S_{O}\right|<20\;\mathrm{dB}$	To avoid saturation of the control signal

**Table 4:** Parameter values for the numerator and denominator polynomials of the proposed 2-DOF $H_{\infty }$ controller.

Parameter	Value	Parameter	Value
$n_{1}$	0	$d_{1}$	0
$n_{2}$	1.315 $\times \;10^{3}$	$d_{2}$	−1.315 $\times \;10^{3}$
$n_{3}$	6.880 $\times \;10^{7}$	$d_{3}$	−6.880 $\times \;10^{7}$
$n_{4}$	8.559 $\times \;10^{11}$	$d_{4}$	−8.559 $\times \;10^{11}$
$n_{5}$	1.544 $\times \;10^{16}$	$d_{5}$	−1.544 $\times \;10^{16}$
$n_{6}$	8.003 $\times \;10^{19}$	$d_{6}$	−8.003 $\times \;10^{19}$
$n_{7}$	2.927 $\times \;10^{23}$	$d_{7}$	−2.927 $\times \;10^{23}$
$p_{1}$	1	$z_{1}$	1
$p_{2}$	1.477 $\times \;10^{5}$	$z_{2}$	1.477 $\times \;10^{5}$
$p_{3}$	1.652 $\times \;10^{9}$	$z_{3}$	1.652 $\times \;10^{9}$
$p_{4}$	3.515 $\times \;10^{13}$	$z_{4}$	3.515 $\times \;10^{13}$
$p_{5}$	1.948 $\times \;10^{17}$	$z_{5}$	1.948 $\times \;10^{17}$
$p_{6}$	7.649 $\times \;10^{20}$	$z_{6}$	7.649 $\times \;10^{20}$
$p_{7}$	1.339 $\times \;10^{22}$	$z_{7}$	1.339 $\times \;10^{22}$

**Table 5:** Tracking errors in terms of the RMSE for the proposed control scheme.

Data	RMSE (μm)		Percentage of Improvement %
	PID-Preisach	Proposed	
A	0.0214	0.0190	11.2
B	0.0267	0.0233	12.7
Mean	0.0241	0.0212	12.03

**Table 6:** Comparison of the improved method with other methods.

Method	Control Structure	Type of PEA	RMSE (μm)
LSSVM [28]	The feedforward–feedback controller was designed by LSSVM without modeling hysteresis.	PEA actuator (T434-A4-201, Piezo Systems, Inc., Cambridge, MA, USA)	0.62
RNN [25]	The feedforward compensator was developed by the deep learning method (RNN).	PEA actuator (P-621.1CD, Karlsruhe, Germany, PI Co.)	0.465
Modified Preisach [52]	The feedforward compensator was developed by the improved inverse Preisach.	PEA actuator (P-885.50, PI Co.)	0.15
PID-NARX-LSSVM [26]	The feedforward compensator was designed by the least-squares support vector machine and the feedback controller was designed by PID.	PEA actuator (name of the company is unavailable)	0.03
PID-Modified Preisach [31]	The feedforward compensator was designed by modified Preisach using PSO-LSSVM and the feedback controller was designed by incremental PID control.	Same actuator (P-752.21C)	0.0241
Fuzzy-PID control [53]	The FB compensator was designed by the fuzzy PI controller.	Same actuator (P-752.21C)	0.333
Modified Bouc–Wen [54]	The nonlinear internal model (estimator) coupled with the Bouc–Wen model for only rate-independent hysteresis.	Same actuator (P-752.21C)	Maximum = 0.015 (for rate-independent hysteresis)
CLC/MRF controller [55]	A complex lead compensator (CLC) using the phase-stabilized compensation method, combined with a multi-resonant filter (MRF).	Same actuator (P-752.21C)	0.0278
The proposed method	The feedforward compensator was designed by modified Preisach using PSO-LSSVM and the feedback controller was designed by the 2-DOF $H_{\infty }$ control.	(P-752.21C)	0.0212

## Data Availability

All data are available in the manuscript.
